# Mechanisms of Phosphorus Removal by Recycled Crushed Concrete

**DOI:** 10.3390/ijerph15020357

**Published:** 2018-02-17

**Authors:** Yihuan Deng, Andrew Wheatley

**Affiliations:** School of Architecture, Building and Civil Engineering, Loughborough University, Loughborough, LE113 TU, UK; a.d.wheatley@lboro.ac.uk

**Keywords:** desorption, recycled concrete, phosphorus removal and recovery, phosphorus speciation by sequential extraction, thermal dynamic, infinite focused microscope

## Abstract

Due to urbanisation, there are large amounts of waste concrete, particularly in rapidly industrialising countries. Currently, demolished concrete is mainly recycled as aggregate for reconstruction. This study has shown that larger sizes (2–5 mm) of recycled concrete aggregate (RCA) removed more than 90% of P from effluent when at pH 5. Analysis of the data, using equilibrium models, indicated a best fit with the Langmuir which predicated an adsorption capacity of 6.88 mg/g. Kinetic analysis indicated the equilibrium adsorption time was 12 h, with pseudo second-order as the best fit. The thermal dynamic tests showed that the adsorption was spontaneous and, together with the evidence from the sequential extraction and desorption experiments, indicated the initial mechanism was physical attraction to the surface followed by chemical reactions which prevented re-release. These results suggested that RCA could be used for both wastewater treatment and P recovery.

## 1. Introduction

Concrete is a major construction material which includes significant embedded resources, these include 50% of all the raw materials used and 40% of the energy. After manufacture, the other 50% of the total waste was generated by the concrete industry [1]. Recently, large volumes of demolition concrete waste have been produced due to limited reuse of older concrete buildings. For example in 2014, China produced 1.5 billion tonnes of construction waste; concrete was 34% of this total and only about 5% was reused [2]. This compares with above 50% in developed countries. New and alternative uses for recycled concrete are a priority in China where, traditionally, construction waste has been disposed of in landfills or deposited on river banks. This disposal used large areas of land as well as causing environmental nuisance and complaints.

There has been previous work on using recycled concrete aggregate (RCA) for water treatment and one of the most researched applications has been as a filter media due to its surface roughness and desirable chemical content (e.g., Mg, Ca, Fe, Al). Li et al. (2007) [3], for example, used fly ash aerated crushed concrete at laboratory scale to achieve 95.6% total nitrogen removal from sewage and landfill leachate. Guo et al. (2009) [4] also used RCA as a wastewater filter and showed it could remove 37% of COD and 55% of total P. The mechanism has been reported to be based on the calcium, aluminium, and iron content which can bind with phosphate [5]. Phosphorus (P) is needed for optimum crop production and mineral phosphorus reserves are thought to be limited with no substitutes. Research in both the U.S. and Europe has shown mineral P resources may be depleted in a few hundred years and recovery of P is urgent to maintain agricultural productivity [6]. Furthermore, removal of P from wastewater to meet environmental quality standards is now a significant cost in chemicals and power, complementing the benefits of recovery, particularly in China [7]. RCA is a lime enriched material, widely available and potentially a sustainable method for P recovery. Other work on P adsorption from wastewater has been reported by Xiang et al. (2013) [5] who suggested RCA could be suitable and sustainable for adsorbing low phosphate concentrations from wastewater. They also reported that the RCA did not re-release P or metals during sludge treatment. RCA has also been used for pH correction of acid wastewaters because of its weakly alkaline properties [8,9]. Instead of using concrete particles, others have used reconstituted concrete blocks. Chen (2001) [10] designed a wastewater treatment system based on concrete blocks. He operated the media as a submerged aerated filter and reported removals of 50% of COD and BOD, 70% of TP, and 20% of TN. Yuan et al. (2006) [11] published results from wastewater using reconstituted concrete blocks, known as eco-blocks which removed 76% of COD and 94.9% of BOD_5_. Japanese researchers have proposed that concrete blocks have greater porosity and provide a larger internal and external surface area for microorganisms [12].

Upon review of the previous literature, there are few well-controlled experiments on the detailed removal mechanisms or reproducibility of P adsorption by either RCA or blocks. Similarly there is little theoretical information on how to design P adsorption systems based on the common adsorption isotherms or reaction kinetics. The aim of this study was to determine the process of P adsorption by RCA. This was achieved by several batch tests with a mechanistic isotherm model. Also, the kinetics and thermodynamics studies were involved to further explain the adsorption mechanism. A novel study of P speciation was carried by sequential extraction. It is first time reported the type of P contained in RCA. This study could contribute to new usage of RCA.

## 2. Materials and Methods

### 2.1. Sorption Studies

Batch experiments were used to evaluate optimum operational conditions for phosphorus adsorption by RCA and data was analysed using adsorption isotherms, kinetics, and thermodynamics. The studies carried out are summarised in Table 1.

**Figure 1:**
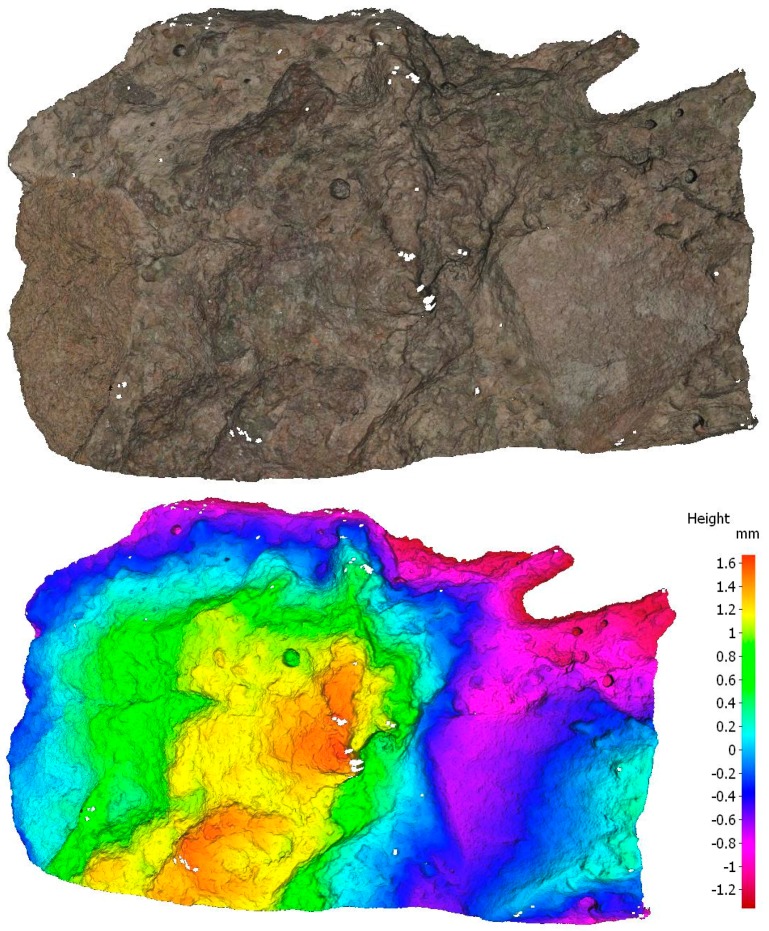
RCA scanning by infinite focused microscope.

Before the experiments, all the glassware and plastic devices were soaked in 10% nitric acid solution for 1 day and then rinsed before each test with 250 mL deionised water. Sieved (2–5 mm) and manually hammered RCA was used for the experiments. RCA was also scanned by an infinite focus microscope (Alicona) (see Figure 1). An infinite focus microscope can quantitatively measure surface texture or deviations in a surface character. Figure 1 shows RCA has a crude surface, which indicates more adsorption sites. The specific surface area of RCA was also identified as 35 m^2^/m^3^. The RCA was examined by EDS (ED3000) and composed of 56% oxygen, 18% Si, 10% Carbon, 10% Ca, 2% Al, 2% K, and 1% Mg. Erhlmeyer flasks, with measured amounts of dried RCA media were used with 100 mL of a known P concentration as a solution of Potassium orthophosphate. The flasks were run in a thermostatically controlled orbital shaker for the defined periods (Table 1). By operating the shaker at various temperatures (Table 1), the data could be used to determine thermodynamic parameters. The pH of the solution was altered using a pH meter (HANNA HI9812-5) with either 0.1 M HCl or NaOH. The final solutions were filtered by a 0.45 µm membrane filter and dissolved concentrations of P were determined by Inductively Coupled Plasma analysis (Shimadzu ICP-9000) according to the international standard method [13]. All tests were conducted in triplicate. The amount adsorbed was calculated using the formula below:

$$q_{e}=\frac{V\left(C_{o}-C_{e}\right)}{W}$$

where $q_{e}$ is the adsorption capacity (mg/g), $C_{o}$ is the initial concentration of solution (mg/L), $C_{e}$ is the concentration after adsorption (mL/L), W is the weight of adsorbent (g) and V is the volume of solution (L).

### 2.2. Desorption of P

The P-treated RCAs from the previous experiments, with 2 g of RCA, were elutriated with 100 mL deionised water in Erhlmeyer flasks. Tests were conducted in triplicate for 24 h mixing and residual P concentration analysed as before.

### 2.3. Fractionation of Inorganic Phosphorus

Sequential extraction to analyse the various P-forms was used to identify the chemical reaction that occurred between the surface of the media and the P solution. Analysis of the P species was first reported by Dean (1938) [14] in soil complexes. Dean defined two types of P according to whether they were extracted by acid or alkali. Chang and Jackson (1957) [15] refined this by using four extraction solutions: loosely bound phosphate (LBP), aluminium phosphate (Al-P), iron phosphate (Fe-P), calcium phosphate (Ca-P), and occluded phosphate (O-P). Most procedures for P characterisation are based on the Chang and Jackson (1957) method with modification or improvements. Normally, Ca and Mg-P are extracted together by HCl [16,17,18]. The Ca-P amount was determined separately and the Mg-P by subtraction. The modification of the Chang and Jackson (1957) [15] and Hartikainen (1979) [19] method was used (see Table 2). After the standard adsorption test, 2 g RCA was used and the analysis was carried out in triplicate.

## 3. Results and Discussion

### 3.1. Sorption Studies

#### 3.1.1. Effect of pH of Solution on Sorption

**Figure 2:**
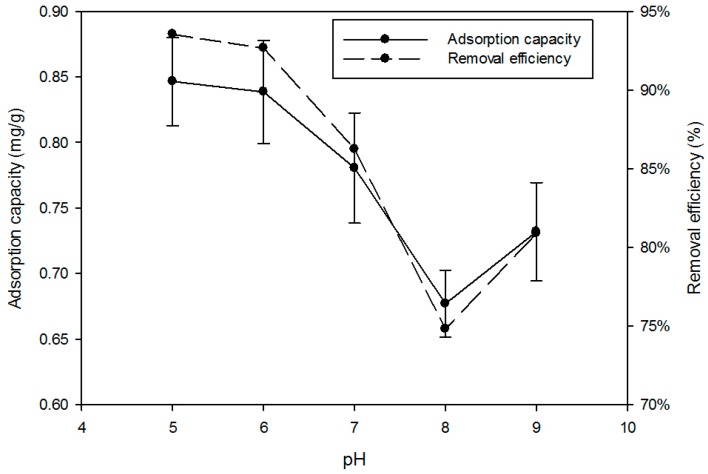
Effect of pH of solution on sorption of P. Dose of media 2 g; initial P concentration 2 mg/L; contact time 24 h; agitator 180-rpm.

The best adsorption was observed at pH 5.0, when the adsorption capacity of P was 0.85 mg/g and removal reached 93.5% (Figure 2). The trend shows a decline in P adsorption with increasing pH to the lowest capacity at pH 8.0. Agyeia et al. [21] also pointed out acidic pH was best for P removal. There are two possible mechanisms, one has been attributed to the double layer effect whereby acidic H^+^ was attracted to the concrete surface by Ca, Al, and Mg hydroxide content creating a secondary positive layer to bind the negative orthophosphate. The second possibility was that the phosphate ions could be converted to their acidic forms (e.g., $H_{2}{\mathrm{PO}}_{4}^{-}$ and ${\mathrm{HPO}}_{4}^{2-}$) binding to the positive surface. The amount of these phosphate ions generated would be proportional to the acidity of the solution. The acidic pH could also help the cement release more Ca^2+^ ions into the solution to react with the hydrogen phosphate causing deprotonation and precipitation of Ca_3_(PO_4_)_2_. The increase in P removal above pH 9 could be due to a similar mechanism but from the formation of OH enriched complexes precipitating calcium phosphate.

#### 3.1.2. Effect of Dose of Sorbent on Phosphorus Sorption

**Figure 3:**
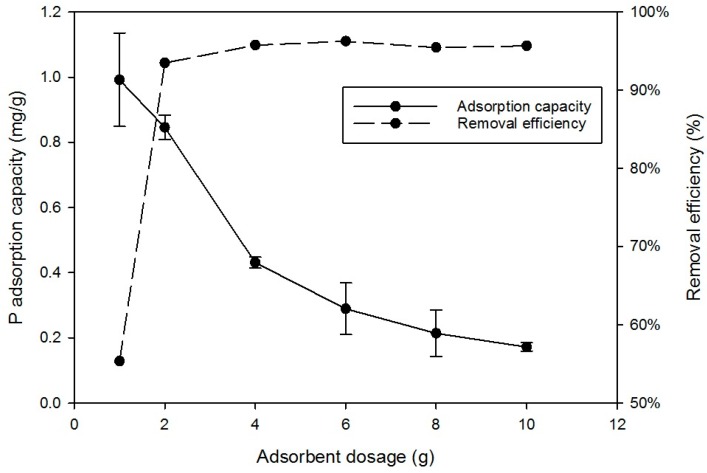
Effect of dose of media on sorption of P. Initial P concentration 20 mg/L; contact time 24 h; agitator 180-rpm; pH-5.

In this experiment, the optimum pH 5 was used and the results in Figure 3 show the amount of P adsorbed was proportional to the amount of RCA added. At the lowest dose (1 g), P removal was 55.5% but 2 g removed 95% of P, similar to the maximum achieved. The normalised adsorption capacity reduced with increasing RCA from 0.99 to 0.17 mg/g, as added RCA was increased from 2 to 10 g. Similar trends have been also reported by other authors [22,23]. Increasing the dose of sorbent increased the total removal of P by increasing the availability of sorption sites. Specific adsorption capacity is a measure of the amount of P bonded by a unit weight of sorbent. The competition by ions for the sites caused a decrease in the specific uptake once all the sites were filled and adsorption capacity decreased with increments in sorbent dose. The simpler models assume a fixed number of adsorption sites according to the molecular structure and the number of positive ions responsible for the binding.

#### 3.1.3. Effect of Initial Phosphorus Concentration

**Figure 4:**
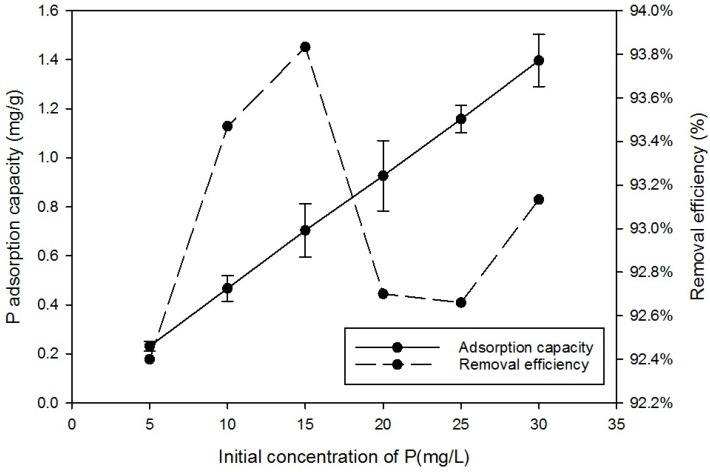
Effect of initial concentration of media on removal of P. Dose of media 2 g contact time 24 h; agitator 180-rpm; pH-5.

Figure 4 shows that the initial concentration of P in the solution influenced the equilibrium uptake of P achieved. The adsorption capacity increased linearly with the initial P concentration from 5 to 30 mg/L, but the proportion of P removed reached maximum at an initial concentration of 15 mg/L. This suggests the simple, single layer, fixed number of adsorption sites model may not be suitable at higher P concentrations. This is in line with previous equilibrium adsorption capacity experiments, which suggest that higher solute concentrations could encourage other mechanisms such as greater boundary concentrations which in turn lead to double layer adsorption and complex formation [23,24].

#### 3.1.4. Equilibrium Studies

The equilibrium equations are shown in Table 3, and were used to determine the mechanisms of adsorption. The Langmuir and Freundlich isotherms gave the best fit (presented in Table 4), the Langmuir showing a maximum adsorption capacity of 6.88 mg/g. The Langmuir is the simplest isotherm, based on monolayer sorption onto a surface with a finite number of identical sites, homogeneously distributed over the sorbent surface. Others have also reported that the Langmuir isotherm was the more robust when modelling complex aqueous mediums. The Frumkin and BET equations were introduced as extensions to the Langmuir isotherm, the Frumkin to take account of solute–solute interaction at a non-ideal surface [25]. The BET was introduced to model multi-layer adsorption but, except for the first layer, it also assumes equal energies of adsorption for each layer and no interactions between layers [26]. The linear correlation of both the Frumkin and BET are weaker than the Langmuir (Table 4). The Freundlich equation included empirical constants as an acceptance that the adsorption sites were heterogeneous. The Temkin and the Dubinin-Radushkevich (D-R) adsorption equations are based on the thermodynamics of adsorption. The Tempkin is based on the principle that adsorption reduces the energy or heat in the adsorbed layer and the D–R on a Gaussian energy distribution as expected from a heterogeneous surface. The D–R model was reported to be suited to high and medium concentration solutions such as these P adsorption experiments [27]. The adsorption energy (E) derived from the D–R model can be used to predict adsorption mechanisms. Typical results are between 1–16 kJ/mol with E values lower than 8 kJ/mol indicating physical sorption [28]. In this study, E was calculated to be 2.24 kJ/mol, which would therefore be considered as physical adsorption. 

Previous filtration studies using cement or concrete have been summarised in Table 5. The majority of these studies used finer particles (>1 mm) to adsorb P because of the greater surface area and consequent adsorption capacity available from smaller sized particles. The aerated concrete had the greatest surface area and gave the largest adsorption capacity. It was reported as too fragile for water treatment however, and small media would also be vulnerable to clogging and wash out, giving high operating costs. The results of this study suggest larger sizes could be used as a compromise for wastewater treatment. The cement studies—by Zheng et al. [32] and Renman and Renman [33]—used a similar size as used here (Table 5) but both materials were unstable. Most of these studies have operated under acidic pH (<3) to achieve maximum P adsorption capacity but these acidic conditions would be unrealistic for most wastewaters. This study has demonstrated that concrete demonstrated reliable P removal at a higher pH (pH 5) which could be achieved in the microenvironment of a wastewater filter.

#### 3.1.5. Kinetic Analysis

Four kinetic models were also used to analyse the data (Table 6). These were the standard first and second-order equations plus the Fractional power equation which is a modified form of Freundlich. Finally the Elovich equation was used since it had previously been used to study ${\mathrm{NH}}_{4}^{+}$ removal by Chien and Clayton (1980) [38]. The results and correlation coefficients (R^2^) are summarised in Table 7. The best fit was pseudo second-order followed by the first order equation. The experimentally measured adsorption capacity (0.749 mg/g) was between those calculated from the pseudo first- and second-order equations. The experimental results indicated that RCA adsorbed most when at high P concentrations; the calculated maximum capacity suggested adsorption could reach 0.9 mg/g.

**Figure 5:**
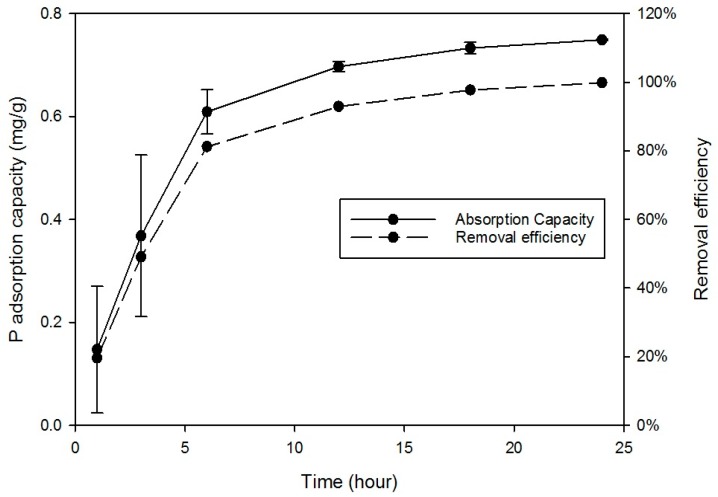
Kinetic studies of P adsorption; Initial P concentration 15 mg/L; agitator 180-rpm; pH-5; temperature = 27 °C.

Figure 5 shows the adsorption rate and removal efficiency increased until equilibrium was reached at 93% P adsorbed after 12 h and 99% at 24 h. The results were carried out at typical domestic wastewater P concentrations (around 10–15 mg/L) and demonstrated a rate of adsorption likely to be achievable in a wastewater treatment plant without excessive costs.

#### 3.1.6. Effect of Temperature on Sorption

The influence of temperature on the thermodynamics of adsorption (Gibbs free energy (ΔG°), Enthalpy change (ΔH°), and Entropy change (ΔS°)) was calculated according to the procedure described by Tosun (2012) [28] as shown in Equations (1)–(3):

(1) ΔG°=−RTb

where R = gas constant (8.314 J/mol K); T = absolute temperature; b = the distribution coefficient, which was calculated by:

(2) $$b=\frac{C_{a}}{C_{e}}$$

where Ca = equilibrium concentration of P on adsorbent (mg/L). The relation between (ΔH°), (ΔG°) and (ΔS°) was given by:

(3) $$\Delta G{}^{\circ}=\Delta H{}^{\circ}-T\Delta S^{{}^{\circ}}$$

**Figure 6:**
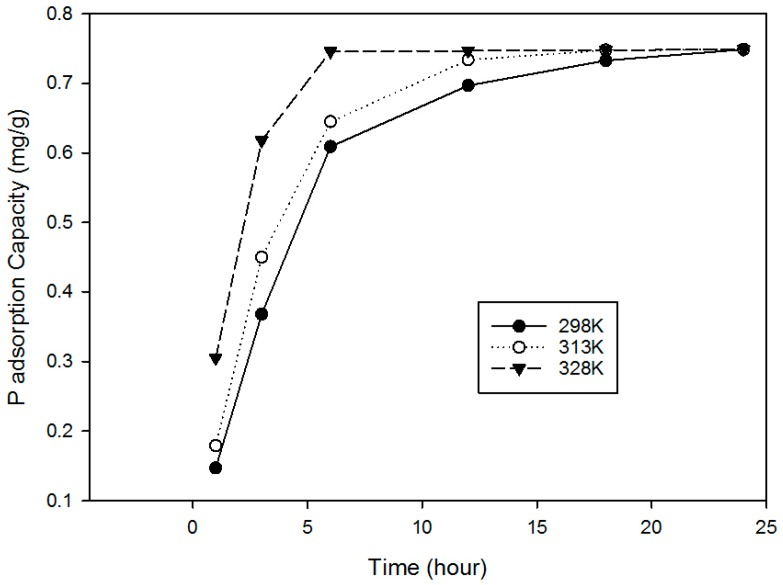
P sorption by concrete studies at various temperatures; Dose of media 2 g; initial P concentration 15 mg/L; contact time 24 h; agitator 180-rpm; pH 5.

**Figure 7:**
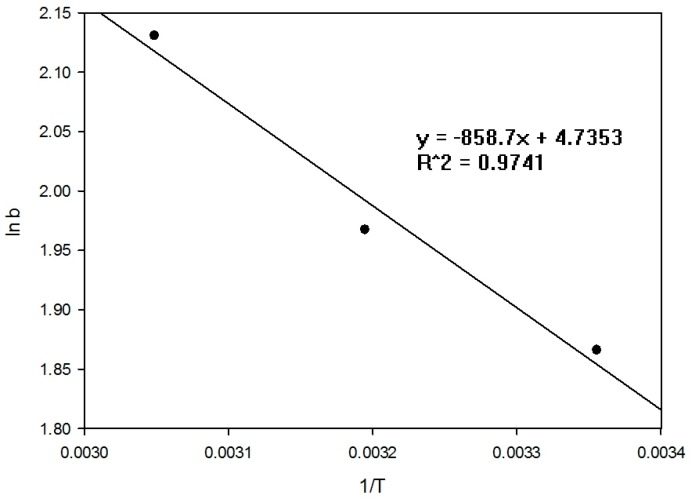
Plot of lnK vs. 1/T for estimation of thermodynamic parameters (ΔH° and ΔS°).

Figure 6 shows that the adsorption of P increased with rises in temperature and that the adsorption process was endothermic. It is suggested that the higher temperature resulted in swelling of the RCA, creating more space for adsorption as well as accelerating molecular diffusion and the chemical reaction. The b value was obtained from $\ln(\frac{Q_{e}}{C_{e}})$ vs. $Q_{e}$ and by extrapolating Q_e_ to zero to obtain the intercept value. Results for ΔG°,ΔH°, and ΔS° were calculated from the slope and intercept of a plot of ln K vs. (1/T) (see Figure 7).

The thermodynamic parameters are summarised in Table 8. The negative values of ΔG° indicate spontaneous adsorption of P on the RCA and the decreasing values of ΔG° confirm the experimental data showing acceleration at higher temperatures. The ΔH° is positive and less than 20 kJ/mol, while the value of ΔH° is less than 20 kJ/mol, indicating the adsorption process is physical [41]. It has been suggested that the positive value of ΔS° is due to the release of the water from phosphate as it is adsorbed onto the surface to increase the degrees of freedom and entropy of the system [42,43]. Other thermodynamic sorption studies for P are summarised in Table 9. The free energy range of RCA was similar to dolomite and activated carbon. Adsorption to these natural materials were also spontaneous, endothermic, and with increased entropy. The similarity to activated carbon also suggests non-specific adsorption sites corroborating the results from the isotherm and kinetic studies.

### 3.2. Desorption of Phosphorus

**Figure 8:**
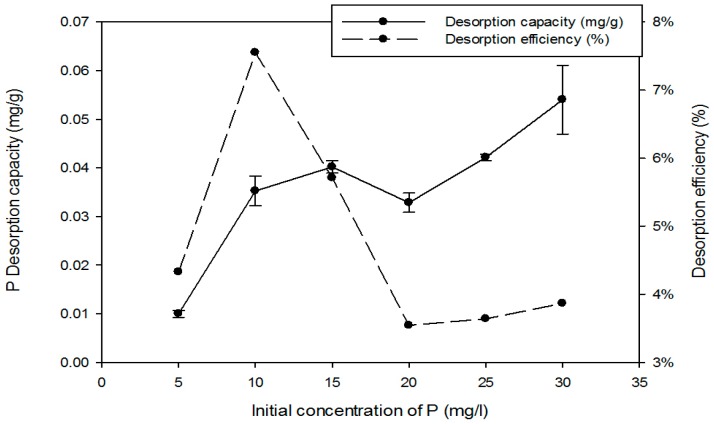
P desorption of concrete on different initial P concentration. Contact time 24 h; agitator 180-rpm; pH-5.

Figure 8 shows the proportion of P re-released. The desorption was in the range of 4–7% and lowest at the higher initial P concentrations with maximum desorption at the initial P = 10 mg/L. The adsorption and desorption capacities were not equal, corroborating a previous hypothesis that different mechanisms were involved at different P concentrations and that some were irreversible. It is suggested that at the lower concentrations adsorption was due to just physical adsorption, which then enabled easier P release. Higher concentrations of P, on the other hand, could be sufficient to cause reactions with the metals such as complexation and precipitation; desorption would therefore be reduced [47]. A lower desorption rate at smaller P concentrations, was also reported by Yu et al. (2009) [35]. They tested desorption from mortar (cement mixed with sand) and found the release was 9% but they deduced that the adsorption was mainly due to chemical reaction.

### 3.3. Fractionation of Inorganic Phosphorus

**Figure 9:**
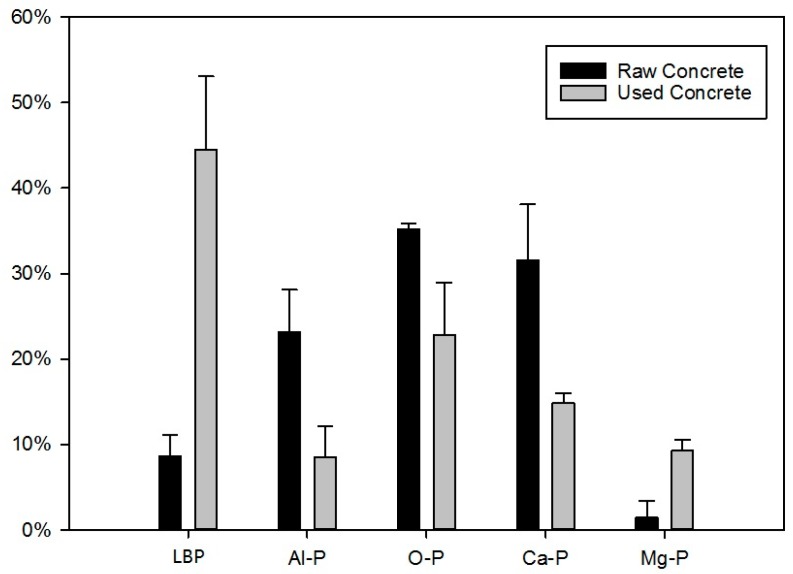
The comparison of different type of P before and after test.

Concrete is composed of Ca, Mg, Si, and Al hydroxides [33,37,48]. The proportions of each metal bound P types were measured. The Si was assumed to be inactive and not analysed. The data is shown in Figure 9: in the raw concrete O-P was the predominant form of P (37%), the remainder was mostly Ca-P (29%) combined with the Al-P, Mg-P and labile P were less than 10%. There was no Fe-P observed in this study. In the used RCA, on the other hand, labile P (43.3%) was the highest proportion, O-P remained high (25%), Al-P and Ca-P were reduced but Mg-P increased, possibly reflecting variations in the mineral composition. Previous work indicates P reacts with calcium and magnesium, from the cement, to form insoluble precipitate (detail see Table 10).

In this study, Mg-P was low in the raw concrete and there are few studies of Mg-P due to its high water solubility. This solubility and reactivity of the Mg-ion compared to Ca, Fe, and Al means Mg-P forms rapidly, but is less crystalline and vulnerable to remobilisation by a wide range of conditions (including pH). Ca-P is more stable due to slower crystallisation [49]. Previous work has also indicated the form of P does vary according to the chemical composition of the filter media (Table 11). Oyster shell, for example, was more than 50% CaO which then formed the majority of the adsorbed P as Ca-P [50]. Zeolite was reported to be 70% SiO_2_, 10% Al_2_O_3_, 2.5% CaO, and 1.5% Fe_2_O_3_ [51,52,53]. The inactivity of Si would explain its poor performance for P removal. In other materials—such as drinking water treatment residues, fly ash, and Bauxite residues—the total P amount was higher than RCA, but the soluble P and sizes available were much smaller, and were not convenient as wastewater filter media.

The used RCA contained 43% soluble P which is the second highest when compared to previous work and blast furnace slag contained most soluble P (see Table 11). Hylander (1999) [58] carried out loading scale pot experiments and stated that slag sorbed P was highly plant available. Therefore, RCA could be used as fertiliser. This is due to the fact that soluble P could be recovered through equilibrium soluble P-concentration, pH change, or redox potential [59,60]. It was confirmed in previous tests that soluble P could be re-released as it was physical attraction, corroborating the predictions of the isotherm, kinetic, and desorption tests. The reaction time was important because previous work has shown that the surface-attracted P might gradually become more permanently bound through complexation and precipitation reactions [54,61]. This was demonstrated in Table 11 which showed adsorption was followed by the formation of apatite complexes. O-P was the second highest indicating that between a third and a quarter of the P became fixed immediately, and would resist re-release, but it still could be re-converted into available P by soil microorganisms (e.g., Phosphate solubilising bacteria) [62]. 

## 4. Conclusions

This study tested recycled concrete for P removal from wastewater, using a larger sized (2–5 mm) and therefore more suitable media than previous studies in practice. More than 90% P removal was achievable using concentrations typical of sewage effluents (*p* < 15 mg/L) with an equilibrium time of 24 h. Model analysis predicted that capacity could reach 6.88 mg/g. The experiments using an analysis of the kinetics (pseudo second-order R = 0.9916) and thermodynamics indicated the adsorption process was spontaneous and, together with the differential extraction and desorption tests results, indicated the mechanism was surface physical electrostatic attraction followed by chemical precipitation. The results suggested that half the P adsorbed to the surface was labile and available for recovery, or, after further crushing, could be used directly as a fertilizer supplement. Further studies on the uptake and impact on plant growth from P enriched RCA would be useful.

The results offer a new usage for high cement RCA which is undesirable for aggregate, due to a lower density, high water adsorption, and reduced structural performance [63,64]. Further work is also suggested to measure how concrete adsorbs heavy metals and persistent organics and how these might interact with C, P, and N in effluents.

## Figures and Tables

**Table 1:** Experimental schedule for the adsorption of P.

No.	Study	Parameters
1	Effect of pH of solution on sorption	The pH of the solution was varied between 6.0 and 8.0. Dose of media 2 g; initial P concentration 20 mg/L; contact time 24 h; agitator 180-rpm
2	Effect of dose of sorbent on phosphorus sorption	RCA was varied in the range 1–10 g, 20 mg/L P solution; contact time 24 h; agitator 180-rpm; pH-5
3	Effect of initial phosphorus concentration	Phosphate concentrations in the range 5–30 mg/L. Dose of media 2 g contact time 24 h; agitator 180-rpm; pH-5;
4	Equilibrium studies	Evaluation of maximum adsorption by isotherm models
5	Kinetic analysis	Initial P concentration 15 mg/L with 2 g of RCA, contact time 24 h at 298 K, agitator 180-rpm, pH 5
6	Effect of temperature on sorption	Initial P concentration 15 mg/L with 2 g of RCA and contact time 24 h at 298, 318 and 328 °C, pH 5

RCA: recycled concrete aggregate.

**Table 2:** Inorganic phosphorus by a sequential extraction for filter media [19,20].

Step	Inorganic P	Extraction Reagents	Concentration	Condition
I	LBP	NH_4_Cl	1 mol/L	50 mL, shaking 0.5 h
II	Al-P	NH_4_F (pH 8)	0.5 mol/L	50 mL, shaking 1 h
III	Fe-P	NaOH	0.1 mol/L	50 mL, shaking 2 h
IV	O-P	CDB (pH 7.6)	-	45 mL, shaking 0.5 h
V	Ca-P	H_2_SO_4_	0.5 mol/L	50 mL, shaking 1 h
VI	Mg-Ca-P	HCl	0.5 mol/L	50 mL, shaking 1 h

Note: CDB (Sodium citrate-sodium dithionite-sodium bicarbonate); Na_3_C_3_H_6_O_7_ (0.3 M); NaHCO_3_ (1 M); Na_2_S_2_O_4_ (1 g).

**Table 3:** Equilibrium study models [25,26,27,29,30,31].

Isotherm Models	Linear Expression	Associated Equations	Parameters
Freundlich	${\mathrm{logq}}_{e}={\mathrm{logK}}_{f}+\frac{1}{n}{\mathrm{logC}}_{e}$	K and n are empirical constants	K_f_ (L/mg) = Langmuir equilibrium constant; n = dimensionless correction factor; qe = adsorption capacity (mg/g)
Langmuir	$\frac{1}{q_{e}}=\frac{1}{q_{m}}+\frac{1}{K_{L}q_{m}C_{e}}$	$R_{L}=\frac{1}{1+K_{L}C_{o}}$	K_L_ = Isotherm constant (L/mg); C_O_ = initial concentration; q_m_=mainxmum adsorption capacity (mg/g)
Tempkin	$q_{e}=B\ln A_{T}+B\ln C_{e}$	-	$A_{T}$ = Tempkin isotherm equilibrium binding constant (L/g); B = constant related to heat of sorption (J/mol)
D-R	${\mathrm{lnq}}_{e}=\ln\left(q_{s}\right)-\left(K_{\mathrm{ad}}\varepsilon^{2}\right)$	$E=\frac{1}{\sqrt{2K_{\mathrm{ad}}}}$	q_s_ = theoretical isotherm saturation capacity (mg/g), K_ad_ = Dubinin–Radushkevich isotherm constant (mol^2^/kJ^2^) and ε = Dubinin–Radushkevich isotherm constant
Frumkin	$\ln\left(\frac{\frac{\theta }{1-\theta }}{C_{e}}\right)=\ln K_{\mathrm{fr}}+2\alpha \theta$	-	θ = fractional surface coverage; α = lateral interaction coefficient; K_fr_ (L/g) = Frumkin equilibrium constant,
BET	$\frac{C_{e}}{q_{e}\left(C_{s}-C_{e}\right)}=\frac{1}{q_{s}C_{\mathrm{BET}}}+\frac{\left(C_{\mathrm{BET}}-1\right)}{q_{s}C_{\mathrm{BET}}}\left(\frac{C_{e}}{C_{s}}\right)$	-	C_e_ = equilibrium concentration (mg/L); C_s_ = adsorbate monolayer saturation concentration (mg/L); C_BET_ = BET adsorption isotherm relating to the energy of surface interaction (L/mg)

**Table 4:** Adsorption Isotherm constants for P adsorption by concrete.

**Langmuir Adsorption Isotherm**				**Freundlich Adsorption Isotherm**					
**q_m_ (mg/g)**	**K_L_ (L/mg)**	**R_L_**	**R^2^**	**n**		**K_f_**		**R^2^**	
6.88	0.089	0.281	0.984	0.996		0.669		0.983	
**Tempkin Adsorption Isotherm**				**Dubinin-Radushkevich Isotherm**					
**A_T_ (L/mg)**	**A_t_**	**B**	**R^2^**	**q_s_ (mg/g)**	**K_ad_ (mol^2^/kJ^2^)**		**ε (kJ/mol)**		**R^2^**
0.195	134.9	0.649	0.958	1.4	2 × 10^−7^		2.24		0.968
**Frumkin Adsorption Isotherm**				**BET Adsorption Isotherm**					
**α**	**K_fr_**		**R^2^**	**C_BET_**		**q_s_**		**R^2^**	
2.959	0.00342		0.669	−0.798		0.553		0.898	

**Table 5:** Adsorptive capacity of various studies by concrete.

Types	Media	Size (mm)	Time	Q_max_ (mg/g)	Reference
Empirical	Ordinary Portland cement	0.045–0.300	16 h	19.90	[21]
Empirical	Recycled Crushed Concrete	0.125–0.250	1 h	0.134	[22]
Empirical	Crushed concrete	0.125	40 days	19.6	[34]
Theoretical	Cement	0.425–0.85	24 h	1.185	[35]
Empirical	Cement	0.85	28 days	16.16	[36]
Empirical	Gas concrete	0.063–2	1 h	11.5	[37]
Theoretical	Recycled crushed concrete	0.3–2.3	24 h	6.1	[23]
Empirical	Crushed autoclaved aerated concrete	2–4	24 h	70.9	[33]
Theoretical	Recycled concrete	2–5	24 h	6.88	Present study
Theoretical	Cement	3–5	32 h	4.98	[32]

**Table 6:** Kinetic study models [39,40].

Kinetic Study	Linear Expression	Parameters
The pseudo-first-order	$\log\left(Q_{e}-Q_{t}\right)=-\frac{k_{1}}{2.303}t+{\mathrm{logQ}}_{e}$	Q = the amount of adsorption time (min) (mg/g); k_1_ = the rate, constant of pseudo first-order sorption (L/min); Q_e_ = adsorption capacities at equilibrium, Q_t_ = adsorption capacities at time t(min)
The pseudo second-order	$\frac{t}{Q_{t}}=\frac{1}{k_{2}Q_{e}^{2}}+\frac{1}{Q_{e}}t$	k_2_ = the rate constant of the second-order equation
Elovich model equation	$Q_{t}=\left(\frac{1}{\beta }\right)\ln\left(\alpha \beta \right)+\left(\frac{1}{\beta }\right)\ln t$	α and β are constants
Fractional power model	$\ln q_{t}=\ln a+b\ln t$	q_t_ = the amount of adsorbate sorbed by adsorbent at a time t; and b = constants with b < 1

**Table 7:** Kinetic parameters for P adsorption on concrete.

**The Pseudo-First-Order**			**The Pseudo Second-Order**		
**k_1_**	**Q_e_**	**R^2^**	**k_2_**	**Q_e_**	**R^2^**
0.211	0.657	0.9876	0.279	0.893	0.9916
**Elovich Model Equation**			**Fractional Power Model**		
α	β	**R^2^**	**a**	**b**	**R^2^**
0.476	5.061	0.9599	0.184	0.5	0.8986

**Table 8:** Thermodynamic parameters for adsorption of P on concrete particles.

Thermodynamic Parameters	Temperature (K)		
	298	313	328
b	6.460	7.150	8.418
ΔG° (kJ/mol)	−4.623	−5.119	−5.808
ΔH° (kJ/mol)	7.139	-	-
ΔS° (J/mol)	39.336	-	-

**Table 9:** Thermodynamic studies for P sorption by other materials.

Adsorbent	ΔH° (kJ/mol)	ΔS° (J/mol)	Reference
Clinoptilolite rich tuff	20.8	100	[44]
Coir-pith activated carbon	3.88	21.88	[41]
Dolomite	−5.85	−10.17	[45]
Granulated ferric hydroxide	15.1	80	[44]
Iron hydroxide-eggshell waste	81.84	-	[46]
Bentonite	−5.3	10	[44]
RCA	7.139	39.336	Present study
Slovakite	104.9	300	[44]

**Table 10:** Formation of P precipitate.

Sequence	Chemical Formula
1	${\mathrm{Mg}}^{2+}+{\mathrm{HPO}}_{4}^{2-}+3H_{2}O\to {\mathrm{MgHPO}}_{4}\cdot 3H_{2}O↓$
2	${\mathrm{Ca}}^{2+}+{\mathrm{HCO}}_{3}^{-}+{\mathrm{OH}}^{-}\to {\mathrm{CaCO}}_{3}↓+H_{2}O$
3	$5{\mathrm{Ca}}^{2+}+4{\mathrm{OH}}^{-}+3{\mathrm{HPO}}_{4}^{2-}\to {\mathrm{Ca}}_{5}\left(\mathrm{OH}\right){\left({\mathrm{PO}}_{4}\right)}_{3}↓+3H_{2}O$
4	${\mathrm{Al}}^{3+}+{\mathrm{PO}}_{4}^{3-}+2H_{2}O\to {\mathrm{AlPO}}_{4}\cdot 2H_{2}O$

**Table 11:** P specification of various saturated material.

Media	Total (mg/g)	Soluble P (mg/g)	Al-P (mg/g)	Fe-P (mg/g)	O-P (mg/g)	Ca-P (mg/g)	Reference
Blast furnace granulated slag	0.086	0.042	-	0.007	-	0.037	[54]
		49%	-	8%	-	43%	
Zeolite	0.448	0.024	0.352	0.041	0.016	0.0131	[20]
		5.4%	78.6%	9.2%	3.6%	2.9%	
Volcanic rock	0.516	0.066	0.311	0.027	0.054	0.058	[20]
		12.8%	60.3%	5.2%	10.5%	11.2%	
Crushed Bricks	0.956	0.068	0.498	0.277	0.024	0.089	[20]
		7.1%	52.1%	29.0%	2.5%	9.3%	
RCA	1.298	0.577	0.110	0	0.297	0.193	Present study
		44.42%	8.50%	0%	22.86%	14.86%	
Oyster shell	3.596	0.363	0.08	0.014	0.589	2.55	[20]
		10.1%	2.2%	0.4%	16.4%	70.9%	
Light-weight expanded clay	6.527	0.053	1.641	0.022	-	4.811	[55]
		1%	25%	<1%	-	74%	
Gas desulfurization products	8.607	2.544	1.452	0.008	0.9	3.703	[56]
		30%	17%	0%	10%	43%	
Bauxite residual	19.487	0.568	14.31	2.203	1.21	1.196	[56]
		3%	73%	11%	6%	6%	
Fly ash	28.074	9.131	16.631	0.147	1.316	0.849	[56]
		33%	59%	1%	5%	3%	
Drinking Water treatment residual	30.031	0.367	20.726	4.66	1.755	2.523	[56]
		1%	69%	16%	6%	8%	
Electric arc fumace steel slag	-	-	-	-	-	-	[57]
		0.63%	3.05%	13.67%	-	82.65%	
Iron melter slag	-	-	-	-	-	-	[57]
		2.41%	22.88%	12.69%	-	62%	
